# Commentary: Evolutionary conservation of acylplastoquinone species from cyanobacteria to eukaryotic photosynthetic organisms of green and red lineages

**DOI:** 10.3389/fpls.2025.1603911

**Published:** 2025-06-04

**Authors:** Naoki Sato

**Affiliations:** Department of Life Sciences, Graduate School of Arts and Sciences, the University of Tokyo, Tokyo, Japan

**Keywords:** acylplastoquinol, cyanobacteria, mass fragmentation, liquid chromatography/mass spectrometry, quantitative analysis

## 1 Introduction

This is a general commentary to the publication by Ito et al. (2025). Plastoquinone serves as a crucial electron carrier in the photosynthesis of cyanobacteria and chloroplasts. Plastoquinone-B (PQ-B) is specifically known for containing an acyloxy group within its prenyl group. Acylplastoquinol (APQ) is an ester of the reduced form of plastoquinone (Mori-Moriyama et al., 2023). The structure of APQ was established by ^1^H- and ^13^C-NMR; however, the determination of its isomeric structure is still pending. APQ has been confirmed by two independent research groups through LC/MS analysis (Ishikawa et al., 2023; Kondo et al., 2023).

In reviewing the paper by Ito et al. (2025), I found some data that need to be re-examined seriously as detailed below.

## 2 Critical examination of the paper

### 2.1 Quality of mass spectral data

**Figure 1:**
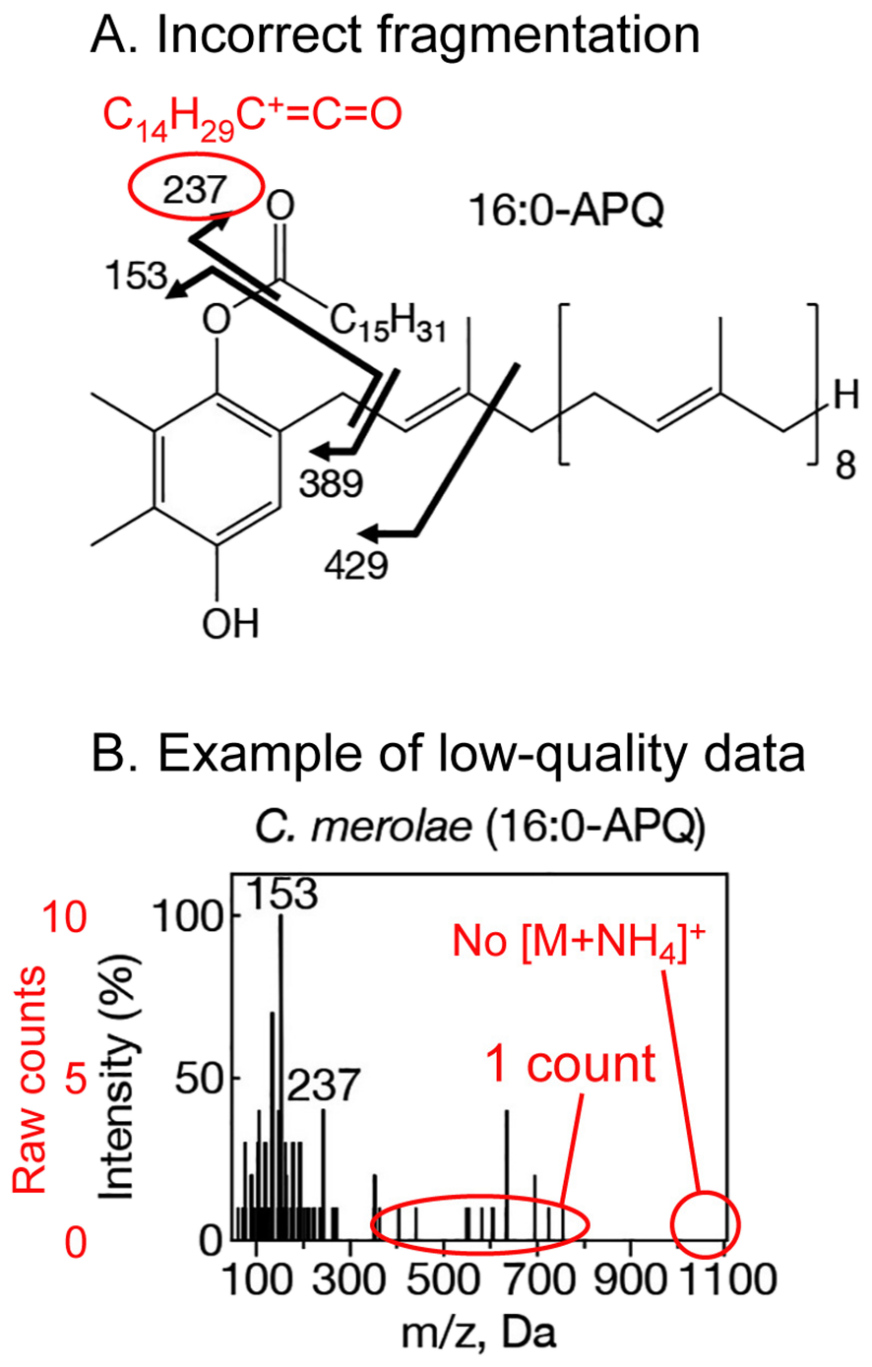
This figure is a part of the work (original **Figure 1**) by Ito et al. (2025) and includes annotations highlighting the points discussed in the text. **(A)** Fragmentation scheme for palmitoyl plastoquinol (16:0-APQ). Note that the *m/z* value of 237 is incorrect for the fragmentation illustrated in the original figure. **(B)** MS/MS spectrum of 16:0-APQ from **(C)** *merolae*. The signal intensity is extremely low, and the parent ion is not detected This figure has been reproduced under the CC-BY license, and the red annotations were added to clarify key discussion points.

**Figure 1B** is an MS/MS spectrum of palmitoyl plastoquinol (16:0-APQ) in *Cyanidioschyzon merolae* presented as **Figure 1** in Ito et al. (2025). The signal intensity was extremely low, as evidenced by the row of low peaks, each representing 1 count, the minimum unit of digital data. The base peak at *m/z* = 153 has only 10 counts. Furthermore, many other spectra (both APQ and PQ-B) exhibit a similar lack of quality. This suggests a very low quantity of the target substance, which could easily be attributed to cross contamination.

Ito et al. (2025) detected primarily saturated APQ, which is also questionable. Mori-Moriyama et al. (2023) and Tanikawa et al. (2025) showed that *Synechocystis* APQ contains both saturated and unsaturated fatty acids. However, Figure 3 of Ito et al. (2025) only presents 16:0- and 18:0-APQ. This discrepancy may arise from the analytical method used. The authors analyzed the total lipid fraction by LC/MS, where each molecular species of APQ appears as separate peaks. The smaller peaks corresponding to unsaturated APQ may be obscured by overlapping peaks from glycerolipids and pigments. Additionally, the fragmentation pattern of unsaturated APQ differs from that of saturated APQ, complicating their detection. It would be more effective to isolate APQ first and then analyze the molecular species using LC/MS.

I found it strange that no [M+NH_4_]^+^ signal was detected in the MS/MS spectra presented by Ito et al. (2025), while a clear [M+NH_4_]^+^ signal was consistently observed in the MS data of *Synechocystis* APQ reported by the same group (Kondo et al., 2023). The signal for the de-prenylated fragment (*m*/*z* = 389 for 16:0-APQ) was either not observed or very weak in the study by Ito et al. (2025). The de-prenylated fragment is crucial in identifying the APQ molecular species, as the acyl fragment is not a reliable marker (see the next section). If the [M+NH_4_]^+^ signal was indeed ionized correctly, we would expect to see the same signal as a prominent parent ion in the MS/MS spectra. Given the very low intensity of the signal and the discrepancies observed, I suspect that the equipment may not have been properly operated. The same argument applies to the MS/MS spectra of PQ-B.

### 2.2 Acyl fragment

The assignment of the acyl-derived fragment in the APQ mass spectrum remains enigmatic. Namely, 16:0-APQ yields a fragment with an *m/z* = 237 (C_14_H_29_C^+^=C=O), rather than the expected 239, which is typically found for the palmitoyl fragment (C_15_H_31_C=O^+^) resulting from esters. Ito et al. (2025) noted that the acyl fragment generated from 16:0-APQ has an *m/z* = 237, but they did not provide an explanation for this assignment (**Figure 1A**, which is adapted from the original **Figure 1** with annotations). Ishikawa et al. (2023) identified this fragment as the RC^+^=C=O ion, yet they also failed to clarify the underlying mechanism. Currently, there is no established explanation for this unusual fragment. The fragmentation scheme in **Figure 1** of Ito et al (2025) will have to be corrected.

### 2.3 Slr2103 orthologs

Ito et al. (2025) stated that the presence of APQ has been demonstrated in only four species of cyanobacteria. However, Tanikawa et al. (2025), who published online prior to the submission of Ito et al. (2025), identified additional cyanobacterial species that also produce APQ, including *Gloeobacter*, which lacks *slr2103* orthologs. Furthermore, while Ito et al. (2025) claimed that *slr2103* orthologs are only conserved within cyanobacteria, Mori-Moriyama et al. (2023) highlighted that the plant PES1, which has an extra domain, is closely related to the cyanobacterial Slr2103 family. This suggests that both plants and algae have the potential to synthesize APQ.

### 2.4 Other points

APQ is an unexpectedly unstable substance that should be handled with caution. Its instability is likely due to its susceptibility to oxidation, which leads to de-acylation. The amount of APQ can decrease during concentration process such as evaporation, drying of thin-layer plates, or other manipulations commonly used in lipid analysis. The low APQ content suspected in the data of Ito et al. (2025), along with the high variability in quantitative data observed in Tanikawa et al. (2025), may result from degradation during these manipulations. It is essential to establish a reliable method for the quantitative analysis of APQ in the future.

## 3 Discussion

APQ is a recently discovered substance that remains challenging to analyze. The analysis of APQ should be conducted with care, utilizing various methods rather than relying solely on a single technique such as LC/MS. In this context, I would like to draw the readers’ attention to a recent paper by Das et al. (2025), which unfortunately did not provide any methodological details or mass spectrometry data. I encourage Ito et al. (2025) to improve their analysis by carefully revising or correcting their initial findings. This would be a constructive step forward in advancing research on the newly identified compounds known as plastoquinone-related lipids.

## References

[B1] Das A. S. Das A. S. Chen Z. Peisker H. Gutbrod K. Hölzl G.. (2025). Multifunctional acylransferases involved in the synthesis of triacylglycerol, fatty acid phytyl esters and plastoquinol esters in cyanobacteria. Planta 261, 123. doi: 10.1007/s00425-025-04700-6 40314852 PMC12048435

[B2] Ishikawa T. Takano S. Tanikawa R. Fujihara T. Atsuzawa K. Kaneko Y.. (2023). Acylated plastoquinone is a novel neutral lipid accumulated in cyanobacteria. PNAS Nexus 2, 1–10. doi: 10.1093/pnasnexus/pgad092 PMC10156143 37152674

[B3] Ito R. Endo M. Aoki M. Fujiwara S. Sato N. (2025). Evolutionary conservation of acylplastoquinone species from cyanobacteria to eukaryotic photosynthetic organisms of green and red lineages. Front. Plant Sci. 16. doi: 10.3389/fpls.2025.1569038 PMC11973298 40196435

[B4] Kondo M. Aoki M. Hirai K. Sagami T. Ito R. Tsuzuki M.. (2023). *slr2103*, a homolog of type-2 diacylglycerol acyltransferase genes, for plastoquinone-related neutral lipid synthesis and NaCl-stress acclimatiza- tion in a cyanobacterium, *Synechocystis* sp. PCC 6803. Front. Plant Sci. 14. doi: 10.3389/fpls.2023.1181180 PMC10171310 37180399

[B5] Mori-Moriyama N. Yoshitomi T. Sato N. (2023). Acyl plastoquinol is a major cyanobacterial substance that co-migrates with triacylglycerol in thin-layer chromatography. Biochem. Biophys. Res. Commun. 641, 18–26. doi: 10.1016/j.bbrc.2022.12.003 36516585

[B6] Tanikawa R. Sakaguchi H. Ishikawa T. Hihara Y. (2025). Accumulation of acyl plastoquinol and triacylglycerol in six cyanobacterial species with different sets of genes encoding type-2 diacylglycerol acyltransferase-like proteins. Plant Cell Physiol. 66, 15–22. doi: 10.1093/pcp/pcae137 39581854

